# Structural and functional abnormalities of penile cavernous endothelial cells result in erectile dysfunction at experimental autoimmune prostatitis rat

**DOI:** 10.1186/s12950-019-0224-0

**Published:** 2019-07-25

**Authors:** Tianrun Huang, Guangchun Wang, Yangyang Hu, Heng Shi, Keyi Wang, Lei Yin, Bo Peng

**Affiliations:** Department of Urology, Shanghai Tenth People’s Hospital, Tongji University School of Medicine, NO 301 Yanchang Road, Shanghai, 200072 People’s Republic of China

**Keywords:** Prostatitis, Erectile dysfunction, Endothelial cells

## Abstract

**Background:**

There is growing recognition of the association of CP/CPPS accompany with ED. However, the specific mechanism of action remains unclear. The aim of this study was to investigate structural and functional abnormalities of cavernous endothelial cells in EAP rat, which may result in the ED.

**Methods:**

we use rat prostate protein extract supplemented with immunoadjuvant to induce EAP rat, ICP and MAP were measured and inflammatory factor infiltration, Akt, eNOS, AR, nNOS and iNOS in the corpus cavernosum were tested. Subsequently, the normal rat and EAP rat cavernosum endothelial cells were purified by MACS, and the metabolism, oxidative stress, MMP, Akt, eNOS, AR and iNOS were evaluated.

**Results:**

The EAP rat model was successfully constructed. The ratio of max ICP/MAP in EAP rat was significantly lower and TNF-α infiltration in corpus cavernosum was significantly higher than normal rats. Besides, Akt, eNOS and AR were decreased, iNOS was significantly increased. The growth and metabolism of endothelial cells in the EAP rats corpus cavernosum decreased and inflammatory factor mRNA was increased and intracellular oxidative stress was also increased significantly. The MMP of EAP rats cavernosum endothelial cells decreased and the expression of Akt, eNOS and AR were also significantly decreased, iNOS was significantly increased.

**Conclusion:**

The prostate suffer local inflammatory infiltrate and promotes cytokines infiltrated into corpus cavernosum caused the oxidative stress increases and the metabolism or MMP decreases. In addition, AR, Akt and eNOS expression and phosphorylation are also reduced, thereby inhibiting the diastolic function of the corpus cavernosum, resulting in decreased erectile function.

## Introduction

Prostatitis is the most common urinary system disease in men under 50 years of age [1]. The clinical manifestations of prostatitis are complex and diverse. Symptoms such as Chronic prostatitis/chronic pelvic pain syndrome (CP/CPPS) are the most common. It is reported that 15% of men experience prostatitis and suffer some symptoms during their lifetime [2], which could significantly reduce their quality of life [3]. With the focus of prostatitis symptoms, more and more studies have shown that there is a correlation between prostatitis symptoms and sexual dysfunction, especially erectile dysfunction (ED). Epidemiological studies suggest that the overall prevalence of sexual dysfunction in patients with prostatitis is between 60 and 75%, and 35–60% of patients have ED or ED couple with other sexual dysfunction [4, 5]. Chung etal found that prostatitis patients were 3.62 times more likely to have ED than general population [6]. Given the high incidence of CP/CPPS in young male populations, so CP/CPPS is considered to be the most common cause of ED in young men [4–6], but the underlying mechanism between prostatitis and ED still unclear.

The penile is consisted of 3 erectile columns, the 2 corpora cavernosa and the corpus spongiosum, as well as the columns’ enveloping fascial layers, nerves, lymphatics, and blood vessels, all covered by skin. The corpora cavernosa contain erectile tissue and are each surrounded by the tunica albuginea, a dense fibrous sheath of connective tissue with relatively few elastic fibers. Along the inner aspect of the tunica albuginea, flattened columns or sinusoidal trabeculae composed of fibrous tissue and smooth muscle surround the endothelial-lined sinusoids (cavernous spaces) [7, 8]. Penile erection is a neurovascular phenomenon that depends upon neural integrity, a functional vascular system, and healthy cavernosal tissues. Nitric oxide (NO), which was produced by endothelial nitric oxide synthase (eNOS) and neuronal nitric oxide synthase (nNOS) under physiological conditions, appears to be the principal neurotransmitter causing penile erection. The release of NO increases the production of cyclic guanosine monophosphate (cGMP), which relaxes cavernosal smooth muscle, leading to arterial inflow increase and the sinusoids within the corpora cavernosa distend with blood. As a result, intracavernous pressure increases and has an erection. At present, the signaling pathways on whether prostatitis participates and reduces erectile function have gradually attracted the attention of scholars. Shoskes et al. found that prostatitis can lead to arterial stiffness associated with NO-mediated endothelial dysfunction [9]. Endothelial dysfunction can inhibit endothelium-dependent vasorelaxation (EDR) [10] and can also strengthen arterial contractions [11]. However, whether prostatitis damage to the corpus cavernosum endothelial cells could causes ED are still unclear.

We have previously established a rat model of experimental autoimmune prostatitis (EAP) to verify the decline in erectile function in EAP rats [12]. On this basis, we concentrate on Structural and functional abnormalities of penile cavernous endothelial cells at experimental autoimmune prostatitis rat.

## Materials and methods

### Experimental animal

Male Sprague-Dawley rat, 6–8 weeks and 180–240 g, was purchased from the animal center affiliated to Nanjing Medical University. All rats breed in constant optimal temperature and humidity for a normal 12-h light and dark cycle. The survey is in line with the “Guidelines for the Care and Use of Laboratory Animals” published by the National Institutes of Health. All procedure approved by the Animal Science Committee of Tongji University.

### Establishment of a rat model of EAP

The EAP model was as previous study reported [12]. Ten rats were used for preparing autologous prostate tissue homogenate supernatant (PTHS). And the rest of 40 rats were randomly divided into EAP model group and control group (20 rats each). In EAP model group, each rat was administered 1.0 mL isovolumetric mixture of PTHS (20 mg/mL) and Freund’s complete adjuvant by multipoint subcutaneous injection; meanwhile, 0.5 mL of a pertussis–diphtheria–tetanus vaccine was performed by intraperitoneal injection. In control group, each rat was injected with isovolumetric PBS instead. After three times of immunizations administered at days 0, 15, and 30, the rat model of EAP was established.

### Assessment of erectile function

At 45th day after the first immunisation, the max intracavernous pressure (ICP) and the ratio of max ICP/mean systemic arterial pressure (MAP) were used to assess erectile function, which have been described previously [12]. The erectile response was elicited by electrical stimulation of the cavernous nerve and quantified by calculating the max ICP/MAP. Rats were anesthetized with intraperitoneal sodium pentobarbital (40 mg/kg, Sinopharm Chemical Reagent Co. Ltd., Shanghai, China). The pressure was measured and recorded using a windows computer programcontrolled multiplying channel physiograph and analyzed using a BL-420 V pressure transducer system (Chengdu Implement Company, Chengdu, China). For each rat, electrical stimulations of the cavernous nerve were stimulated at a frequency of 12 Hz and using a pulse width of 5 msec. Stimulations were performed in triplicate at 5 V for 30 s with intervals of 5 min between subsequent stimulations. The mean max ICP/MA*P* values were considered to represent the erectile function of rats.

### Inflammatory infiltration of rat prostate and corpus cavernosum

Rats was sacrificed at 45th day after the first immunisation (about 87–101 days). Rats were euthanized using intraperitoneal sodium pentobarbital (150 mg/kg) and immediately remove the penis and prostate tissue. A portion of the corpus cavernosum and prostate tissue was fixed overnight in 4% paraformaldehyde, and the remainder was stored in liquid nitrogen for further analysis. The fixed tissue was then dehydrated in 70% ethanol and embedded in paraffin. These paraffin-embedded samples were cut into sections, and then deparaffinized and subjected to HE staining. After dyeing for 15 min in the hematoxylin dyeing tank, the running water was slightly washed with hematoxylin 1–3 s, 1% hydrochloric acid ethanol for 1 min, double distilled water for 10–30 s, and quickly placed in 0.5% eosin for 3 min, double steaming. The water is rinsed and dehydrated.

Immunohistochemistry of TNF-α, after washing three times with PBS, 50–100 μl of normal goat serum was added dropwise, incubated at room temperature for 20 min until blocking, and then the first antibody was dropped into a wet box and incubated for 2 h. After washing three times with PBS, 50 μl of enhancer was added and incubated in a wet box for 30 min at room temperature. The secondary antibody was incubated for 30 min after washing three times with PBS. It was then washed with PBS and stained with DAB, stained with hematoxylin for 10 min and dehydrated and sealed. The expression of TNF-α in the tissues was observed under a light microscope.

### Measuring oxidative stress levels

SOD activity, MDA and NO levels were used to evaluate oxidative stress state. The SOD activity, MDA and NO levels was measured by using commercial kits (Nanjing Jiancheng Bioengineering Company, Nanjing, China) following the manufacturer’s instructions.

### Isolation and purification of corpus cavernosum endothelial cells

The cavernous endothelial cells of normal control rats and EAP rats were isolated and purified by enzymatic digestion and magnetic activated cell sorting (MACS). Rats were anesthetized by intraperitoneal injection of 3% pentobarbital sodium. After incision of the foreskin and fascia, removal of tissues such as the urethra, dorsal veins and artery, the penis was quickly cut and submerged in pre-cooled phosphate buffered saline containing 100 μg/mL penicillin and streptomycin, and rinsing and removal of blood coagulation Piece. The penile tissue was then placed under a microscope (Nikon, Model C-DSD230, Japan) to remove the tunica albuginea, and the corpus cavernosum was cut into small pieces (1 mm^3^). Subsequently, 200 U/mL collagenase IV (GIBCO, 17104019, USA) was added to Hanks Balanced Salt Solution containing Ca^2+^ and Mg^2+^ (Invitrogen, 14025092, USA) and digested on a 37 °C shaker. Minute. The digestive juice containing the cells is then collected and replenished with fresh collagenase IV-containing digestive juice until the digestive corpus cavernosum is completely digested. Add 0.25% trypsin (GIBCO, 15050065) and 1 mg/mL DNase I (Rubio, D3212, China) without EDTA in the collection of collagenase IV containing cells, continue digestion for 5 min, and then add 20% fetal The digestion was terminated by EGM-2 medium (LONZA, cc-3156 & cc-4176, USA) of bovine serum (FBS; GIBCO, 10270106, South America). The digestive juice containing the cells is filtered through a sieve to separate into individual cells. The cell pellet was collected by centrifugation at 300 x g for 10 min, and the cells were resuspended and cultured in EGM-2 medium at 37 °C, 5% CO_2_. The culture flask was pre-coated with 1% gelatin.

When the cells grow to 80% confluence, the old culture medium in the cell culture flask is aspirated by a pipette, the cells are washed with PBS solution, 0.5 ml of 0.05% trypsin solution containing EDTA is added, and the digested cells are observed under an inverted microscope. After the cells are retracted, the cells are no longer connected to the tablets, and then the serum-containing medium is added to completely terminate the digestion, and then the cell suspension is gently blown. After centrifugation at 200 g for 10 min, the supernatant was removed and the cell pellet was collected. The cell pellet was washed again with Buffer and subjected to cell counting. 10 μL of PE mouse anti-rat CD31 (BD, 555027, USA) was added to each 107 cells for 15 min at 4 °C. After centrifugation as above, 20 μL of anti-PE MicroBeads (Miltenyi Biotec, 130–048-801, Germany) was added. Then, after incubating for 15 min at 4 °C, the cells were washed again, resuspended in 500 μL of buffer, and poured into a separation column (Miltenyi Biotec, 130–042-201) placed in a magnetic field for sorting. The CD31+ cells were sorted and adsorbed on the separation column, while the CD31- cells were eluted with the buffer. After removing the separation column from the magnetic field, the CD31+ attached to the tube wall was quickly washed with 1 mL of buffer, and the purified corpus cavernosum endothelial cells were obtained. Purified corpus cavernosum endothelial cells were added to EGM-2 medium and cultured at 37 °C and 5% CO_2_ .

### Identification of endothelial cells by immunofluorescence staining

Purified corpus cavernosum endothelial cells were cultured on coverslips placed in Millicell (Millipore, PEZGS 0816, USA). At 80–90% confluence, cells were fixed in paraformaldehyde (4%) for 30 min, then permeabilized with Triton X-100 for 20 min and blocked with 5% BSA for 1 h. Cells were incubated with primary antibodies including anti-CD31 (ab119339, 1:400), anti-vWF (ab6994, 1:400), Anti-CD90 / Thy1 (ab92574, 1:200), anti-Desmin (ab32362, 1:50) overnight at 4 °C and then incubated for 2 h in fluorescein-labeled secondary antibodies. Room temperature. Subsequently, the nuclei were stained with 4,6-diamidino-2-phenylindole and observed by confocal microscopy (ZEISS LSM700).

### Cell growth and metabolism testing

Cell growth rate was measured using CCK-8. The cell pellet was collected, washed again with the culture medium and subjected to cell counting. Add 100 μl of cell suspension containing the corresponding cell number to each well of a 96-well plate, set up five replicate wells in each group, and culture the 96-well culture plate in 37 °C, 5% CO_2_ cell incubator until the cells are attached. Discard the old culture medium, add 200 μl of the complete broth of the corresponding inflammatory factor per well, and incubate at 37 °C in a 5% CO 2 cell culture incubator. After 24 h, 48 h, 72 h, 10 μl of CCK-8 solution was added to each well, and air bubbles were avoided as much as possible. The culture was continued for 2 h at 37 °C in a 5% CO_2_ cell incubator and then determined by a microplate reader at 450 nm. The absorbance at the place.

### Flow cytometry for cell purity

The cells were harvested and resuspended in 100 μL of PBS containing 107% BSA and 2 mmol/L EDTA per 10^7^ cells. 10 μL of PE mouse anti-rat CD31 was added to the suspension, and the cells were incubated for 10 min at 4 °C in the dark. Finally, the cells were washed, resuspended in 500 μL of buffer and analyzed by flow cytometry (BD, inflow, cell sorter, Franklin Lakes NJ, USA).

### Flow cytometry of changes in mitochondrial membrane potential (MMP)

MMP was measured using fluorescent dye JC-1 (Yeasen, 40706 ES60, Shanghai). Briefly, after washing the corpus cavernosum cells of normal control rats and EAP rats with PBS, JC-1 was added to the cultured cells for 30 min at 37 °C in the dark. Fluorescence intensity was estimated using BD FACS Canto II at 485 nm excitation and 590 nm emission. CCCP treated cells lasted for 20 min as a positive control.

### Detection of TNF-α, IL-1β, IL-6 and AR, eNOS and AKt mRNA in ESP rat corpus cavernosum endothelial cells by RT-qPCR

Total RNA was extracted from tissue samples using TRIzol and concentrations and mass were measured. The mRNA was synthesized into cDNA and used as a template for qPCR. The reaction consisted of 10 μL of 2X Real-time PCR Master Mix, 2 μL of each primer and 7 μL of EPC water. The reaction conditions were as follows: pre-denaturation was carried out at 95 °C for 5 min, denaturation at 40 °C for 15 s, annealing at 60 °C for 20 s, and elongation at 72 °C for 40 s. The primer sequences are shown in Table 1.

**Table 1:** The primer sequences. The GADPH was used as an internal control

Item	5′---------3’
TNF-α-F	AGGGAATTGTGGCTCTGGGT
TNF-α-R	AGGCCACTACTTCAGCGTCT
IL-1β-F	AGAATGGGCAGTCTCCAGGG
IL-1β-R	GACCAGAATGTGCCACGGTT
IL-6-F	ATTCTGTCTCGAGCCCACCA
IL-6-R	AGGCAACTGGCTGGAAGTCT
AR-F	CCAGGGACCATGTTTTGCC
AR-R	CGAAGACGACAAGATGGACAA
Enos-F	GTCTGGAGGGCTAAGCAGTC
Enos-R	GCAAGGAAGGTTGACAGTATGC
Akt-F	GTGGACTTACCTTATCCCCTCA
Akt-R	TTGGCTTTGGTCGTTCTGTTT
GAPDH-F	GCAAGTTCAACGGCACAG
GAPDH-R	GCCAGTAGACTCCACGACAT

### Western blot analysis of AR, eNOS and AKt expression in EAP rat corpus cavernosum endothelial cells

For protein extraction, cells were washed once with PBS prior to lysis. Next, 250 mL of ice-cold modified RIPA buffer (Beyotime Institute of Biotechnology, Shanghai, China) was used to contain protease and phosphatase inhibitors using a cell scraper, and the cells were collected into EP tubes. Lysates were sonicated for 20 s (25% power, 0.5 cycles), centrifuged at 12,000 xg for 30 min at 4 °C, and the clarified supernatant was transferred to a new tube. Protein concentration was determined using a BCA assay (Beyotime Institute of Biotechnology).

Western blotting was performed under standard conditions. Approximately 40 mg of protein lysate was separated on a 10–12% SDS-PAGE gel and transferred to a PVDF membrane. The PVDF membrane was then blocked with 5% milk for 2 h at room temperature and incubated with the primary antibody overnight. Phosphorylated eNOS (ab184154,1:500) and anti-eNOS (ab76198, 1:1000), anti- inducible nitric oxide synthase (iNOS) (ab3523, 1:500), anti-nNOS (ab5586, 1:500), Anti-CD90/Thy1(ab92574,1:1000),anti-Desmin(ab32362, 1:5000) were obtained from Abeam; anti-AR (sc-7305,1:200) was obtained from Santa Cruz Biotechnology; phospho-Akt (4060,1:1000) and Akt antibody (9272,1:1000) were obtained from Cell Signaling Technology. Rabbits or mouse antibodies conjugated to horseradish peroxidase (HRP) in the dark after washing for 4 times in Tris buffered saline containing 0.1% Tween 20 for 15 min (Thermo, 34160 and 31430) Incubate for 2 h. Protein bands were detected by using an Enhanced Chemiluminescence system (Beyotime Institute of Biotechnology), then autoradiographed and quantified by densitometry. GAPDH (sc-32233, 1:5000) was used as an internal reference to normalize the data.

### Statistical analysis

All statistical analyses were performed by using SPSS 20.0 (SPSS, Inc., Chicago, IL, USA). Data were presented as mean ± standard deviation. Differences between groups were analyzed by using an unpaired Student’s t-test. A value of *p* < 0.05 was considered statistically significant.

## Results

### Histopathological features of EAP rats

**Fig. 1:**
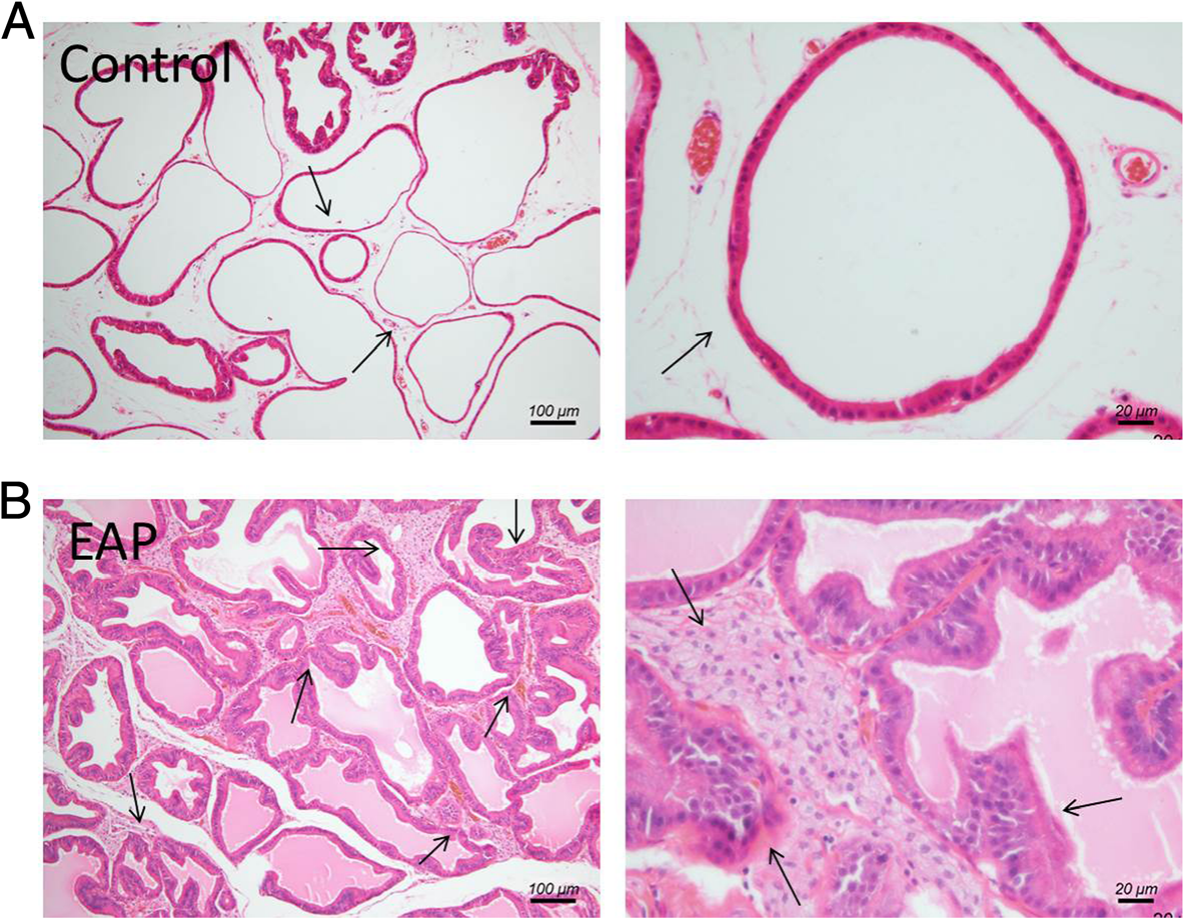
Histopathological features of ventral prostate tissue in control and EAP rats. In the normal rat, the glandular epithelium structure of prostate glands was integrated and clear without inflammatory cells infiltration and tissue hyperplasia (**a**). In EAP rat, the prostate duct is irregularly shaped and diffusely inhomogeneous hyperplasia, partial basal lamina was infiltrated by chronic inflammatory cells (**b**). EAP: Experimental autoimmune prostatitis

Rat ventral prostate was examined. As shown in Fig. 1, in normal control rat (A), the glandular epithelium structure of prostate glands was integrated and clear without inflammatory cells infiltration and tissue hyperplasia. In EAP rat (B), the prostate duct is irregularly shaped and diffusely inhomogeneous hyperplasia, partial basal lamina was infiltrated by chronic inflammatory cells. The prostatic histopathological features of the experimental prostatitis rat model in this study are consistent with the diagnostic criteria for simulating human CP/CPPS [12].

### Effect of CP/CPPS on erectile function in rats

No statistically significant difference was observed in the total body weight as well as the penis weight among EAP rats and controls. Lower max ICP/ MAP ratio was observed in the EAP group as compared to the control group. Show in Table 2.

**Table 2:** Comparisons of body and penis weight, erectile function in two groups

Item	EAP	Control	*P* value
Body weight (g)	510.10 ± 9.76	515.93 ± 8.90	0.080
Penis weight (mg)	374.09 ± 9.22	369.05 ± 8.95	0.901
Max ICP (mmHg)	64.83 ± 8.16	94.33 ± 4.76	0.001*
MAP (mmHg)	133.78 ± 10.31	125.67 ± 4.82	0.263
Max ICP/MAP	0.48 ± 0.03	0.75 ± 0.02	0.001*

*p < 0.05 Max ICP Max Intracavernosal Pressure, *MAP* Mean Arterial Pressure

### Infiltration of TNF-α and AR, eNOS, nNOS and Akt changes in EAP rats

**Fig. 2:**
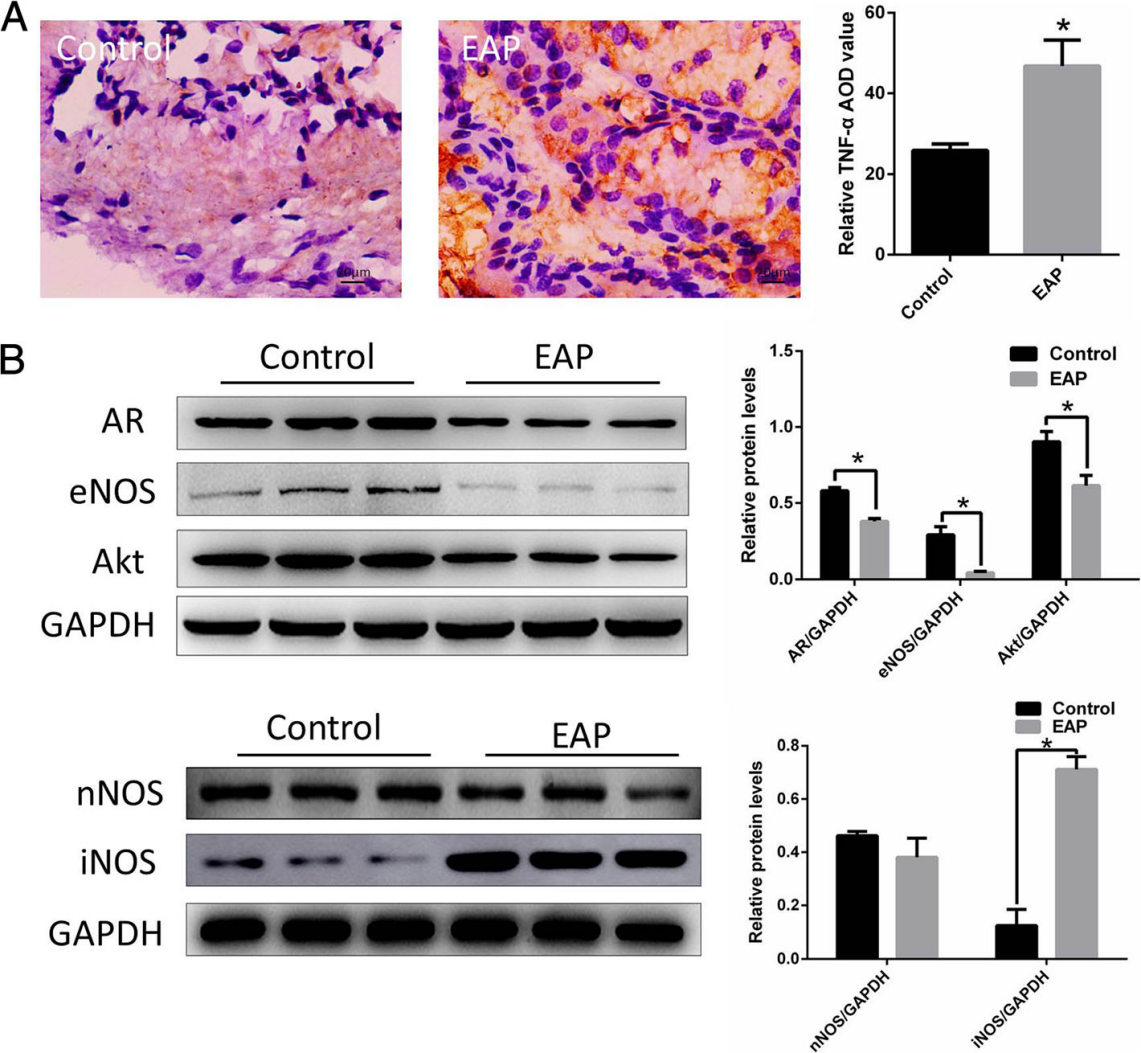
The degree of infiltration of TNF-α in the corpus cavernosum of EAP rats was significantly higher than that of normal rats (**a**). The expression of AR, eNOS and Akt in the corpus cavernosum of EAP rats decreased significantly, iNOS increased significantly (**b**). *p < 0.05. EAP: Experimental autoimmune prostatitis, AR: Androgen receptor, eNOS: endothelial nitric oxide synthase, iNOS: inducible Nitric-Oxide Synthase

As shown in Fig. 2, The degree of infiltration of TNF-α in the corpus cavernosum of EAP rats (A right) was significantly higher than that of normal rats (A left), and the expression of AR, eNOS and Akt in the corpus cavernosum of EAP rats was significantly decreased, iNOS was significantly increased (as show in Fig. 2b, *p < 0.05).

### Identification of cavernous endothelial cells

**Fig. 3:**
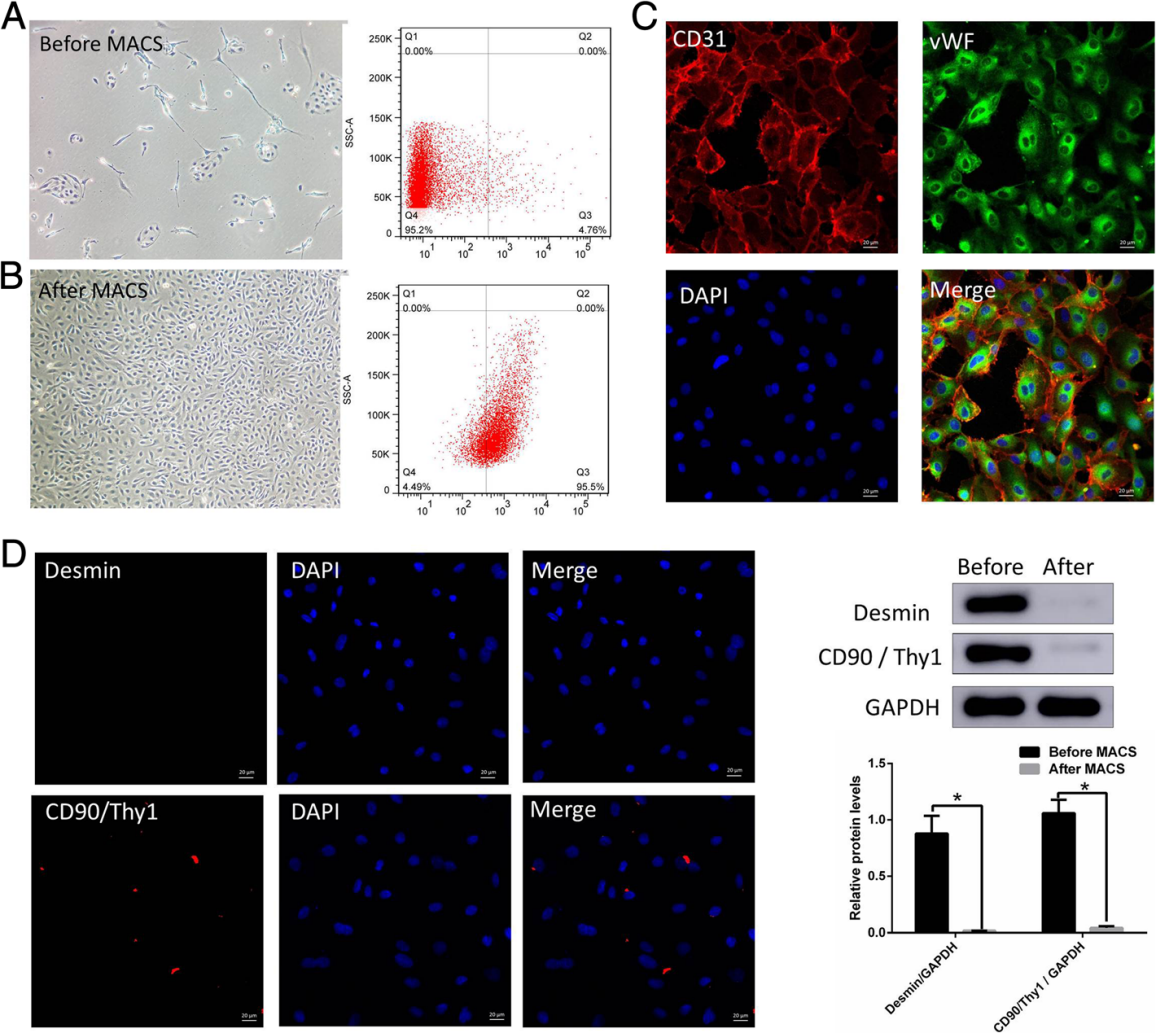
Purification and identification of cavernous endothelial cells. The cobblestone-like morphology of the corpus cavernosum endothelial cells (**a** and **b**). Before the sorting by immunomagnetic beads, a large number of lumps or long spindles were formed to form fibroblasts and smooth muscle cells (**a** left). Flow cytometry showed that 5.81 ± 0.17% of the cells were CD31 + (**a** right) before purification. And the proportion of CD31 + after MACS increased significantly to 95.32 ± 0.38% (**b** left). Almost all cells were CD31 positive (red) in the cell membrane and vWF positive (green) in the cytoplasm (**c**). Immunofluorescence (left) and Western blotting (right) confirmed cells after MACS lack the expression of Desmin and CD90 / Thy1 which were strongly expressed in smooth muscle and fibroblast cells (**d**). *p < 0.05. MACS: Magnetic-activated cell sorting VWF: Von Willebrand factor

The corpus cavernosum endothelial cells maintained as cobblestone-like morphology phenotype (Fig. 3a and b). Before being sorted by immunomagnetic beads, a large number fibroblasts and smooth muscle cells mixed with s cavernosum endothelial cells (Fig. 3a). Flow cytometry showed that 5.81 ± 0.17% of the cells were CD31 + (Fig. 3a right) before purification. And the proportion of CD31+ after MACS increased significantly to 95.32 ± 0.3811% (Fig. 3b right). It was further confirmed by immunofluorescence (Fig. 3c) that almost all cells observed were CD31 positive (red) in the cell membrane and vWF positive (green) in the cytoplasm. Immunofluorescence and Western blotting confirmed cells after MACS lack the express of Desmin and CD90/Thy1 which were strongly expressed in smooth muscle and fibroblast cells (Fig. 3d).

### Changs of cell growth and oxidative stress in rat corpus cavernosum endothelial cells

**Fig. 4:**
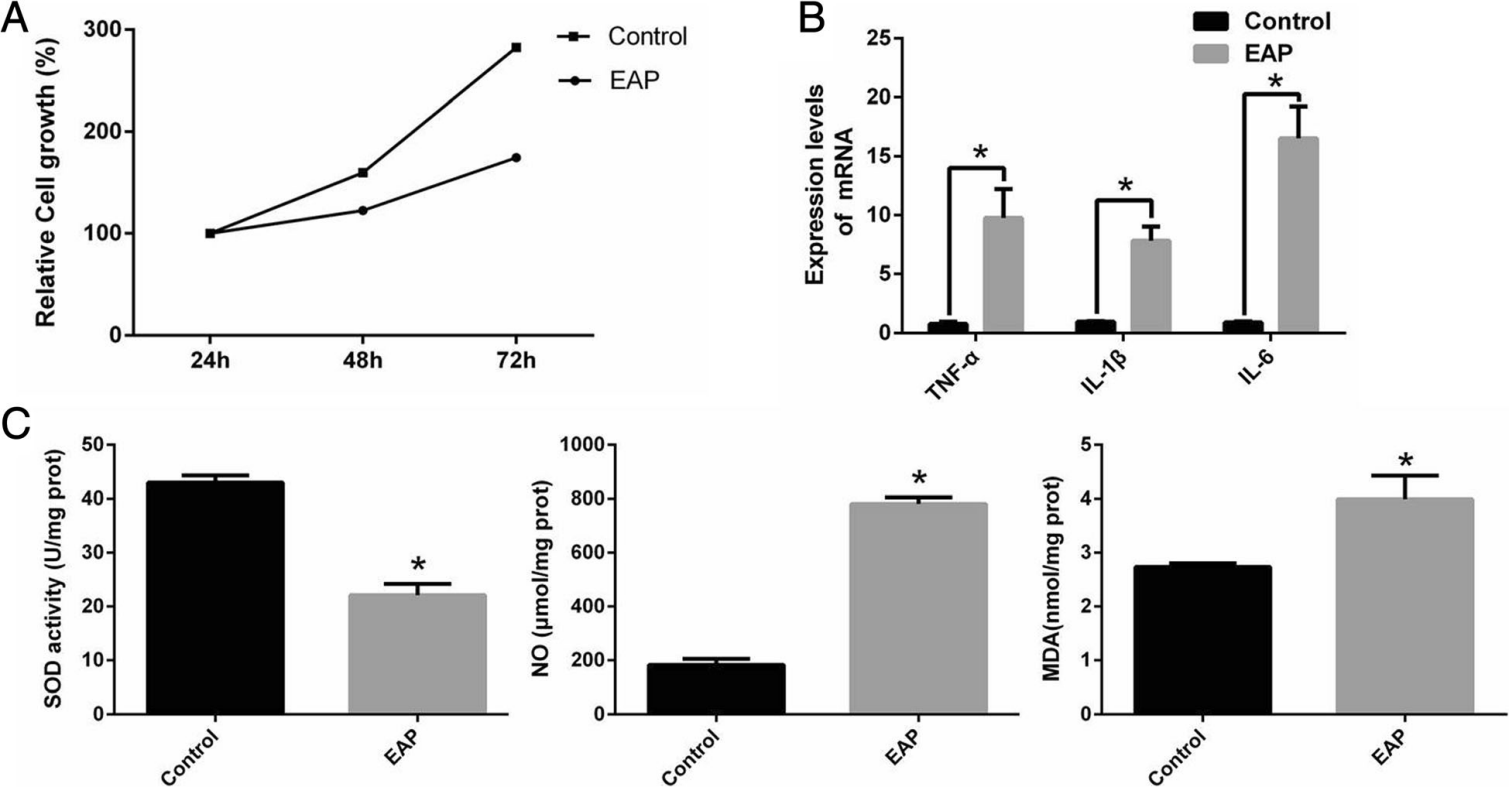
EAP rat corpus cavernosum endothelial cells growth rate decreased, oxidative stress increased. **a**: The EAP rat corpus cavernosum endothelial cells showed shows a significant decrease in relative cell growth within 72 h compared with the normal control. **b**: The mRNA levels of TNF-α, IL-1β and IL-6 in EAP rat corpus cavernosum endothelial cells were significantly increased compared with normal controls. **c**: NO and MDA levels were significantly increased and SOD activity was significantly decreased in EAP rat corpus cavernosum endothelial cells. *p < 0.05. EAP: Experimental autoimmune prostatitis NO: Nitric oxide SOD: Superoxide Dismutase MDA: Malondialdehyde

Compared with the normal control, the EAP rat corpus cavernosum endothelial cells showed a significant decrease in relative cell growth within 72 h (Fig. 4a). After 72 h of culture, the mRNA levels of TNF-α, IL-1β, and IL-6 in EAP rat corpus cavernosum endothelial cells were significantly increased compared with normal controls (Fig. 4b). And the NO level in the EAP rat corpus cavernosum endothelial cells was 781.0 ± 14.19 μmol/mg prot, the MDA level was 3.993 ± 0.22 nmol/mg prot, which was significantly higher than that of the normal control rat corpus cavernosum endothelial cells (NO: 182.7 ± 13.20 μmol/mg prot, MDA: 2.736 ± 0.02 nmol/mg prot); SOD level in EAP rat corpus cavernosum endothelial cells was 22.08 ± 1.06 U/mg prot, significantly lower than that of normal control rat corpus cavernosum endothelial cells (42.97 ± 0.68 U/mg prot) (Fig. 4c).

### Changs of mitochondrial membrane potential level in rat corpus cavernosum endothelial cells

**Fig. 5:**
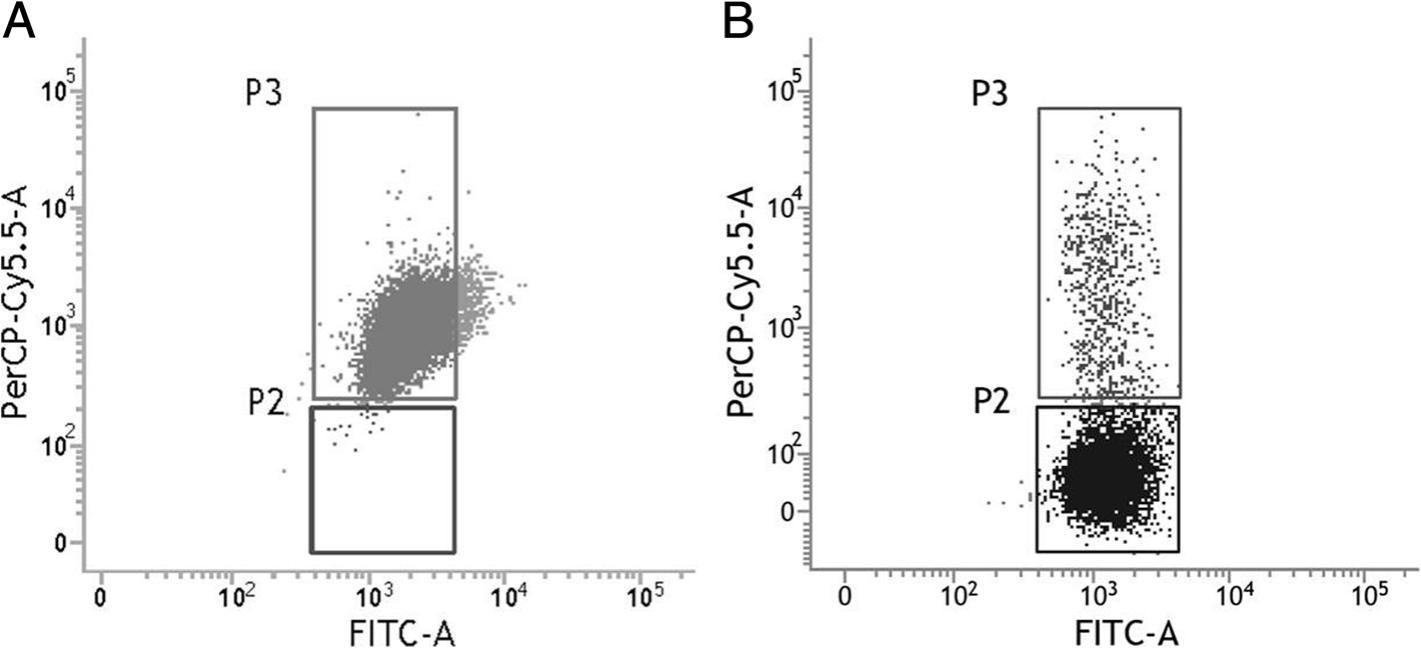
The mitochondrial membrane potential of EAP rat corpus cavernosum endothelial cells was significantly decreased. **a**: normal control rat corpus cavernosum endothelial cells. **b**: EAP rat corpus cavernosum endothelial cells

As shown in Fig. 5, Flow cytometry showed that 90.46 ± 0.97% cells in EAP rat corpus cavernosum endothelial cells (B) and only 0.39 ± 0.19% of normal control rat corpus cavernosum endothelial cells (A) were in P2, indicating EAP rats mitochondrial membrane potential level was significantly decreased.

### AR, eNOS, AKt and iNOS in EAP rat corpus cavernosum endothelial cell decreased significantly

**Fig. 6:**
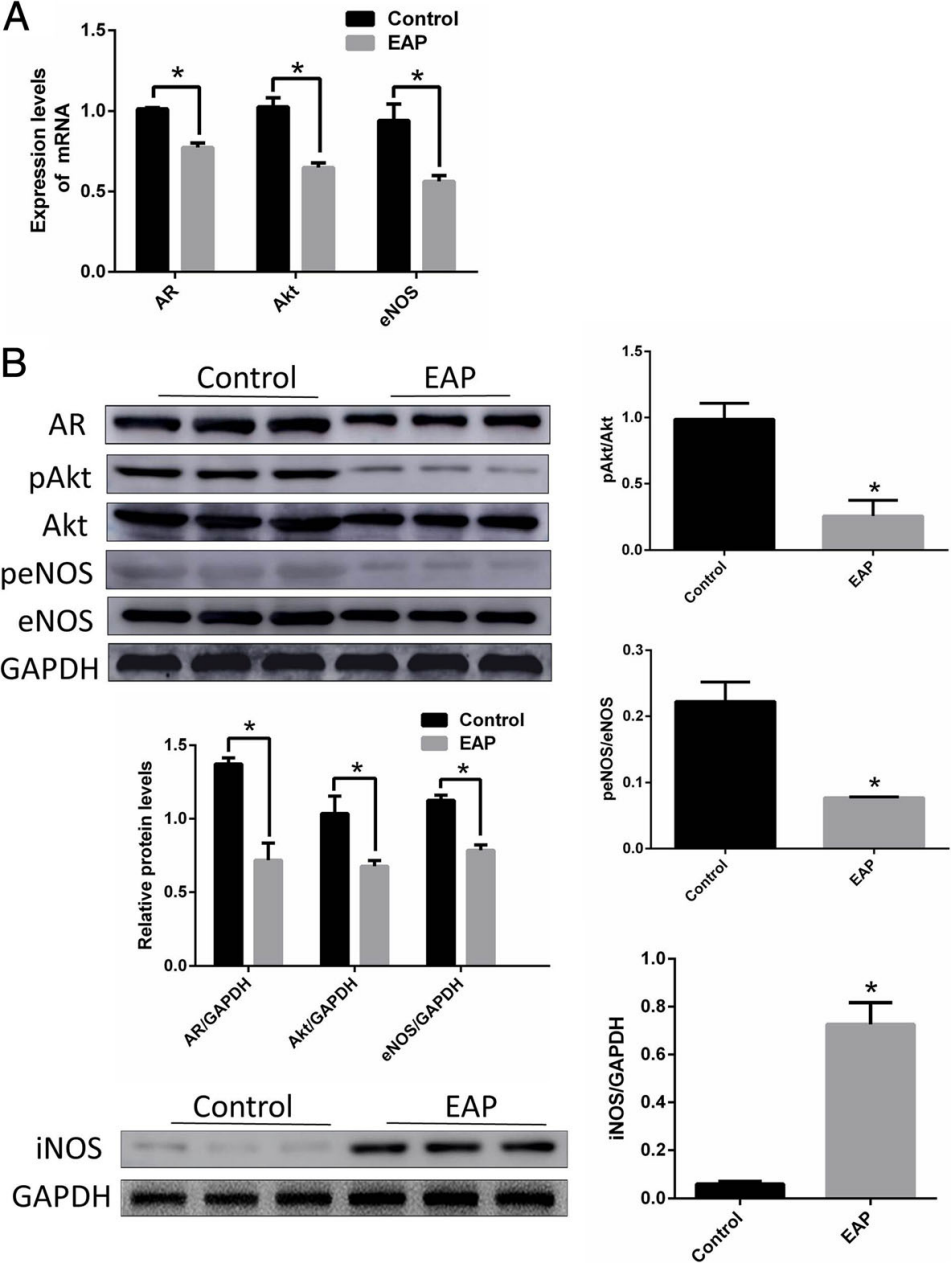
Expression of AR, eNOS, AKt mRNA and protein in corpus cavernosum endothelial cell. **a**: The expression level of AR, eNOS and AKt mRNA were significantly decreased in EAP rat corpus cavernosum endothelial cell group compared with control. **b**: The expression level of AR, eNOS and AKt protein were significantly decreased, and iNOS were significantly increased in EAP rat corpus cavernosum endothelial cell group compared with control. And Western blot was normalized to GAPDH. *p < 0.05. AR: Androgen receptor EAP: Experimental autoimmune prostatitis eNOS: endothelial nitric oxide synthase iNOS: inducible Nitric-Oxide Synthase

As shown in Fig. 6, the levels of AR, eNOS and AKt mRNA (Fig. 6a) and protein (Fig. 6b) were significantly reduced in EAP rat cavernous endothelial cells compared to control rat. In addition, pAkt and peNOS were significantly decreased, indicating that phosphorylation of Akt and eNOS were inhibited. iNOS protein was significantly increased in EAP rat cavernous endothelial cells.

## Discussion

In recent years, the correlation between chronic prostatitis and ED has also received increasing attention. Many epidemiological studies have suggested that the incidence of ED in patients with chronic prostatitis is significantly higher than in the normal population [13, 14] and patients with chronic prostatitis with ED are also associated with lower urinary tract symptoms [4]. Previous studies have suggested that psychological disorders caused by prostatitis play a significant role in causing ED [15–17], such as CP / CPPS induce pain, lower urinary tract symptoms, and have a negative impact on the sex life [18], but organic damaged caused by CP/CPPS was rarely reported.

Endothelial cells play an initial role in the erectile process through the eNOS-NO-cGMP pathway [19], it is important to successfully isolate and compare the differences in endothelial cell function in chronic prostatitis. Since the first report of the isolation of penile cavernous endothelial cells by tissue block implantation in 1989 [20], several methods have been applied [21] and more currently used elastic elastase digestion [8]. Theoretically, elastase does not decompose the extracellular matrix,so avoid mixing smooth muscle cells and fibroblasts. In practice, due to tissue shearing and mechanical compression, substrates are exposed to make a large number of “spindle” smooth muscle and fibroblasts. In this study, we improved the use of collagenase IV to remove the corpus cavernosum cells from the white membrane. Collagenase IV contains low pancreatin activity, which slightly digests multiple tissues and minimizes cell membrane damage. The CD31 labeled immunomagnetic beads were used for sorting. CD31, also known as Platelet endothelial cell adhesion molecule-1 (PECAM-1), is expressed in tight junctions between cell membranes such as vascular endothelial cells and platelets, and endothelial cells. vWF is synthesized and secreted by vascular endothelial cells, which are mainly expressed in the cytoplasm of endothelial cells. Desmin and CD90/Thy1 which were strongly expressed in smooth muscle and fibroblast cells. In this study, we found that CD31 and vWF were highly expressed by cell immunofluorescence, and CD31 was mainly expressed on the cell membrane, VWF was mainly expressed in the cytoplasm, which also met the corresponding protein localization of CD31 and vWF. And Western blotting showed cells after MACS lack the band of Desmin and CD90 / Thy1, which means MACS discharged the mixed smooth muscle and fibroblast cells effectively.

In this study, we first constructed an EAP rat model, and the prostate histopathology of EAP rats met the diagnostic criteria for simulating human CP/CPPS. In the previous study, Hu etal has reported a significant increase in inflammatory mediators and corpus cavernosum oxidative stress in EAP rats, and the erectile function of EAP rats is significantly reduced [12]. In this study, we further examined the expression of androgen receptor, Akt, and endothelial nitric oxide synthase in the corpus cavernosum of EAP rats. Compared with normal control rats, Akt and endothelial oxidative in EAP rats. The down-regulation of nitrogen synthase and androgen receptors suggests a decrease in the function of penile cavernous endothelial cells in prostatitis rats.

In this study, we found that the levels of SOD and MDA in EAP purified endothelial cells were significantly higher than control. SOD is superoxide dismutase, which can specifically remove free radicals in the body to relieve the damage caused by free radical oxidation; MDA is Malondialdehyde, which is the final metabolite of lipid oxidation, causing toxic stress in cells. The electrophilic reaction reflects the degree of lipid oxidation of the cell membrane [22], the decrease in SOD level and the increase in MDA levels clearly reflect the increased degree of oxidative stress in the corpus cavernosum and endothelial cells.

Increased TNF-α infiltration was showed in EAP rat corpus cavernosum. We also found the mRNA levels of TNF-α, IL-1β and IL-6 in EAP corpus cavernosum endothelial cells were also significantly increased, and the metabolism of endothelial cells is significantly reduced. TNF-α can increase monocyte-macrophage capacity, activate NF-κB pathway to mediate inflammatory cascade, promote adhesion of various adhesion factors such as ICAM-1, and promote adhesion between neutrophils and endothelial cells [23], IL-1β is one of the most potent inflammatory factors. As a pro-inflammatory cytokine, IL-1β aggravates pain, edema and other reactions by promoting the expression of COX-2, iNOS and ICAM, and interacting with various inflammatory factors [24].

NO synthesized and released from cavernous endothelial cells by eNOS is an important factor in penile erection, which involved Akt-eNOS-cGMP pathway [25]. In this study, we reported eNOS was decreased significantly both in EAP rat corpus cavernosum and corpus cavernosum endothelial cells combined with lower max ICP/ MAP ratio. Akt is a serine/threonine protein kinase that also regulates phosphorylation of e-NOS Ser1177 in endothelial cells to produce NO, activates guanylate cyclase, and subsequently synthesizes cGMP, which activates cGMP-dependent calcium ions. The pathway reduces intracellular calcium ions, resulting in relaxation and erection of cavernous smooth muscle cells. eNOS phosphorylation is an important step in the production of NO by eNOS after protein translation. The down-regulation of phosphorylation of Akt and eNOS inhibitied endothelial-dependent vasodilation and aggravating the degree of vasoconstriction and sclerosis [10, 11, 26]. In this study, we found iNOS get significant increased both in EAP rat corpus cavernosum and corpus cavernosum endothelial cells, and the level of NO in the corpus cavernosum endothelial cells of EAP rats was elevated, which was mainly caused by iNOS synthesis. In addition to eNOS, iNOS can also produce NO, but iNOS is not expressed in normal physiology, and its expression is usually induced by pathological stimulation such as cytokines. And unlike eNOS, which is mainly involved in the synthesis of physiologically optimal levels of NO and maintains normal physiological functions of endothelial cells, iNOS is almost 6–10 times more active than eNOS. iNOS rapidly produces high levels of NO when stimulated by various inflammatory stimuli [27]. A large amount of NO reacts with superoxide to produce peroxynitrite, causing lipid peroxidation and DNA. Cytotoxic reactions such as damage lead to cell and tissue damage [28]. Anti-inflammatory treatment could reduced the expression of iNOS in human umbilical vein endothelial cells [29], and endothelial dysfunction get ameliorated via inhibiting iNOS expression [30].

Mitochondria are the main synthetic organelles of ATP and are important sites for energy supply in the body. Synthetic ATP is exchanged into the cytoplasm by ADP/ATP vectors in the mitochondrial inner membrane and ADP in the cytoplasm. During the process of respiratory oxidation, mitochondria store the energy generated by electrochemical potential energy in the mitochondrial inner membrane, causing asymmetric distribution of proton and other ion concentrations on both sides of the inner membrane to form mitochondrial membrane potential. Normal MMP is a prerequisite for maintaining mitochondrial oxidative phosphorylation and producing ATP. The stability of MMP is beneficial to maintain the normal physiological function of cells. The decrease of MMP marks the abnormality of cell energy metabolism and is the earliest signal of apoptosis [31]. Wang XJ and other scholars used projection electron microscopy to observe the corpus cavernosum of chronic nonbacterial prostatitis rats, and found a large number of mitochondria to moderate to severe swelling, and the mitochondrial inner membrane and outer membrane were destroyed [32]. Under the condition of oxidative stress, the oxygen free radicals can be promoted. When SOD can not effectively remove these oxygen free radicals, the excess oxygen free radicals can directly or indirectly damage the mitochondrial membrane, causing the MMP to decrease, leading to the inhibition of ATP synthesis. Metabolism eventually accelerates apoptosis. In this study, the Mitochondrial membrane potential (MMP) of penile cavernous endothelial cells was significantly reduced.

Previous studies reported that there is no significant changes in serum testosterone levels in CP/CPPS [12, 32, 33]. Our study found that AR decreased in EAP rat and in corpus cavernosum endothelial cell, and AR expression reduced greatly reduce the bioavailability of androgens [34]. Khalili M etal found a significant decrease in AR expression in infected prostate glands by preparing a bacterial prostatitis model [35], and very low TNF-α and IL-1β exposure can also lead to inhibition of the androgen receptor pathway [36, 37]. The AR antagonist flutamide, can significantly inhibit the proliferation, migration and colony formation of endothelial cells [38]. Testosterone and dihydrotestosterone combined with AR can up-regulate the expression of VEGF-A, cyclin A, and cyclin D1 to promote endothelial cell proliferation and help repair endothelial cells in the inflammatory environment [39].

## Conclusion

The prostate suffer local inflammatory infiltrate and promotes the release of cytokines infiltrated into corpus cavernosum. Besides,the oxidative stress increases and the metabolism or MMP decreases significantly. In addition, AR, Akt and eNOS expression and phosphorylation are also reduced, thereby inhibiting the diastolic function of the corpus cavernosum, resulting in decreased erectile function.

## Data Availability

Please contact author for data requests.

## Abbreviations

AR: Androgen receptor
cGMP: Cyclic guanosine monophosphate
CP/CPPS: Chronic prostatitis/chronic pelvic pain syndrome
DAB: Diaminobenzidine
EAP: Experimental autoimmune prostatitis
ED: Erectile dysfunction
EDR: Endothelium-dependent vasorelaxation
eNOS: endothelial nitric oxide synthase
ICAM: Intercellular cell adhesion molecule
ICP: Intracavernosal pressure
iNOS: inducible Nitric-Oxide Synthase
MACS: magnetic activated cell sorting
MACS: Magnetic-activated cell sorting
MAP: Mean arterial pressure
MDA: Malondialdehyde
MMP: Mitochondrial membrane potential
nNOS: neuronal nitric oxide synthase
NO: Nitric oxide
PTHS: Prostate tissue homogenate supernatant
SOD: Superoxide Dismutase
VWF: Von Willebrand factor
